# Impact of institutional quality improvement initiatives on metabolic monitoring in mental disorder in patients treated with antipsychotics: A meta-analysis of intervention studies

**DOI:** 10.7189/jogh.14.04074

**Published:** 2024-05-24

**Authors:** Sooyoung Shin, Suhyeon Moon, Jua Wang, Yeo Jin Choi

**Affiliations:** 1 Department of Biohealth Regulatory Science, Graduate School, Ajou University, Suwon, Republic of Korea; 2 Department of Pharmacy, College of Pharmacy, Ajou University, Suwon, Republic of Korea; 3 Research Institute of Pharmaceutical Science and Technology, Ajou University, Suwon, Republic of Korea; 4 Department of Pharmacy, College of Pharmacy, Kyung Hee University, Seoul, Republic of Korea

## Abstract

**Background:**

Compliance with guidelines regarding monitoring of metabolic adverse effects induced by antipsychotics has been consistently low. We characterised and evaluated the quality of institutional quality improvement (QI) interventions designed to address disparities between guidelines and real-world practices. Furthermore, we assessed the impact of these interventions on the screening and management of metabolic risks for inpatients receiving treatment with antipsychotic medications.

**Methods:**

We conducted a meta-analysis of institutional QI intervention studies aimed at improving antipsychotic-associated metabolic risk monitoring in hospitalised mental disease patients. Relevant studies were identified through searches conducted in the Embase and PubMed databases, as well as by reviewing previous reviews and meta-analyses. Quantitative analyses were performed, calculating odds ratios (ORs) and 95% confidence intervals (CIs) to assess the impact of QI programmes on guideline adherence in clinical practice.

**Results:**

We identified 12 intervention studies (n = 10 128 and n = 2667 patients in the pre-and post-intervention groups, respectively) and included them in our meta-analysis. QI interventions demonstrated effectiveness in bridging the guideline-practice gap in monitoring antipsychotic-induced metabolic adverse effects, as supported by the ORs and 95% CIs for post-intervention monitoring of plasma glucose, lipids, and blood pressure (BP) vs the pre-intervention period being OR = 6.90 (95% CI = 1.51–31.48), OR = 5.39 (95% CI = 4.01–7.24), and OR = 4.81 (95% CI = 1.23–18.79), respectively. Only 33.3% (4/12) of studies reported screening rates for all four metabolic parameters (plasma glucose, lipids, weight/body mass index (BMI), and BP). The median rates for metabolic screening of plasma glucose, lipids, and BP increased from 51.0–80.0%, 28.7–66.7%, and 91.7–95.8%, respectively. Up to 66.7% (8/12) of intervention studies lacked follow-up measures to treat or manage identified risks in hospitalised psychiatric patients, such as patient referrals, prescription of medications, and switching of antipsychotics. The odds of monitoring weight/BMI and glucose were greatest when QI programmes involved the participation of multidisciplinary health care professionals and patients, yielding OR = 3.35 (95% CI = 2.45–4.59) and OR = 57.51 (95% CI = 24.11–137.21), respectively.

**Conclusions:**

Institutional QI interventions were effective in enhancing monitoring practices in alignment with established guidelines for metabolic risk screening among hospitalised patients with mental disorders maintained on antipsychotic medications. Future institutional QI programmes should incorporate multidisciplinary strategies involving patient engagement and extend their focus beyond screening to incorporate follow-up risk management strategies once risks have been identified.

**Registration:**

PROSPERO CRD42023452138.

Patients with severe mental illness (SMI) are at increased risk for cardiovascular (CV) and metabolic dysfunctions due to their disease states, as well as antipsychotic therapy known for inducing metabolic adverse effects [1–4]. Second-generation antipsychotics (SGAs) are characterised by lower risks of extrapyramidal symptoms-related side effects compared to first-generation antipsychotics [5,6]. However, greater risks of hyperglycaemia and hyperlipidaemia have been associated with SGAs in several studies, thereby predisposing SMI patients with long-term SGA exposure to elevated risk of cardiometabolic complications [7–10].

To optimise health outcomes in SMI patients, it is essential to screen for metabolic adverse effects potentially induced by their antipsychotic therapy. Several clinical guidelines also recommend that antipsychotic medication users are to be monitored for possible metabolic abnormalities in terms of plasma glucose, lipid, prolactin, and thyroid stimulating hormone (TSH) levels, along with changes in blood pressure (BP), electrocardiogram (ECG), weight, body mass index (BMI), and waist circumference [11–19]. However, despite the publication and communication of guideline recommendations, many mental disease patients using antipsychotic medications are still left unscreened for cardiometabolic complication risks [20–22]. A previous meta-analysis also reported that the effects of the guideline publication on metabolic risk screening rates among these high-risk patients, albeit substantially improved, remained suboptimal. The uptake of guideline recommendations on screening for glucose and lipid levels was observed only in 56% and 29% of antipsychotic-treated patients, respectively [23].

A prior study showed that the provision of guidelines and passive observation of guideline compliance in clinicians’ practice led to a positive, albeit still suboptimal, effect on improving metabolic monitoring in high-risk SMI patients [23]. However, there is still a gap between guideline recommendations and real-world clinical practice with respect to overall screening rates and subsequent interventions in case of abnormal metabolic findings in these patients [23]. In response to these findings, several quality improvement (QI) initiatives aiming to enhance screening for antipsychotic-associated metabolic risks have been implemented in clinical practice both in community and inpatient settings [24–26]. A previous systematic review on these QI studies reported that, although the included studies contained several limitations in terms of study design, quality, and outcome measures, overall metabolic monitoring rates in SMI patients further improved with the implementation of QI interventions in clinical practice [27].

SMI patients typically present with multiple barriers to health care access; hence, an inpatient admission may pose an opportunity for health care professionals to screen for CV and metabolic risks potentially associated with antipsychotic medication therapy of these patients [28,29]. There has been no meta-analysis on the efficacy of institutional QI initiatives to promote cardiometabolic screening, primarily in inpatient settings, where substantial impact can be achieved with the implementation of such interventions among antipsychotic-treated SMI patients. Therefore, we aimed to review and characterise QI intervention studies conducted with the intention of improving cardiometabolic risk screening per clinical guidelines among adult inpatients treated with antipsychotic therapy and evaluate the quality and efficacy of intervention strategies employed in individual studies.

## METHODS

### Data sources and search strategy

We utilised the Preferred Reporting Items for Systematic Reviews and Meta-analyses (PRISMA) to guide our methodology and to prepare this manuscript [30]. We registered a protocol in the International Prospective Register of Systematic Reviews (PROSPERO), registration number CRD42023452138.

Relevant articles of institutional QI studies on increasing metabolic monitoring rates among hospitalised patients with mental illness treated with antipsychotic medications, published from inception to June 2023, were identified through electronic searches by using search terms in Medline/PubMed and Embase databases (last date searched was 27 October 2023). The initial database search terms are pre-specified as follows: (antipsychotic or psychi* or psycho* or bipolar or schizophr* or mental or mood or depression or severe mental illness) in title/abstract AND (monitor* or screen* or exam* or test*) in title/abstract, (weight or glucose or lipid or BP or metabolic) in title/abstract, and (inpatient or hospital*) in title/abstract.

Reference lists of previous reviews were manually screened for any missed original articles. Only published, peer-reviewed articles in English were included in the study analyses. Two authors (SS and YJC) independently performed the title/abstract search, and then a full-text review to identify the articles satisfying inclusion criteria of the current study. Any disagreements between authors were resolved by discussion and consensus. Ethics committee review and informed consent from study participants were waived as this meta-analysis was conducted by pooling existing patient data extracted from published primary research.

### Criteria for study selection

Studies that satisfied the inclusion criteria specified below and made available monitoring rates in the followed-up patients before and after the implementation of QI interventions were found eligible and selected for data extraction. The inclusion criteria were as follows: studies evaluating routine metabolic monitoring practices in adult inpatients with mental illness, prescribed antipsychotic agents, and studies investigating the impact of QI interventions to improve the metabolic screening practices in inpatient settings. Antipsychotic medications included both first- and second-generation agents. Those studies with insufficient data, such as conference abstracts, were excluded from study analyses. Metabolic risk screening practices should have involved at least one of the four categories of metabolic measures: 1) weight, BMI, or waist circumference, 2) glucose – fasting blood glucose or haemoglobin A1c (HbA1c), 3) lipids – total cholesterol, low-density lipoprotein cholesterol, high-density lipoprotein cholesterol, or triglyceride, and 4) BP – systolic BP, or diastolic BP.

Studies were required to have an experimental or pre-post intervention design in which QI programmes to enhance monitoring of SMI patients treated with antipsychotics for metabolic adverse drug reactions were implemented into hospital-based clinical practice. Those studies that examined the effect of passive uptake of monitoring guidelines on metabolic screening rates among antipsychotic users but with no institutional interventions implemented into clinical practice were excluded from this review.

In cases where metabolic screening rates were reported at several time points, the final time point available was selected as the outcome for study analysis. In uncontrolled pre-post designs, the post-intervention group was considered the intervention group, whereas the pre-intervention group was the control group. As guideline recommendations are only accounting for antipsychotic agents and not applicable to psychotropic agents, studies examining metabolic screening of patients taking selective serotonin reuptake inhibitors or mood stabilisers were excluded.

### Data extraction

Data extraction was performed by one author (SS) and checked by a second author (YJC) using a pre-determined data extraction form. Demographic information of study patients (age, gender, and ethnicity), psychiatric diagnoses, antipsychotic medications, intervention types, study design, audit schedules, sample size, and metabolic measures examined, along with their monitoring rates in the pre- vs post-intervention groups, were extracted from the included studies. Additionally, intervention strategies employed in individual studies to enhance metabolic monitoring in hospitalised psychiatric patients treated with antipsychotics were extracted for study analysis.

### Risk of bias and quality assessment

We utilised the Effective Public Health Practice Tool, which can be applied to quantitative studies with randomised and non-randomised designs, to evaluate the quality and risk of bias of the included studies [31]. The studies were appraised with regard to the six components of selection (two items), design (three items), confounders (two items), blinding (two items), data collection, and withdrawals and dropouts (two items); 11 items in total. Each component is scored: one for strong, two for moderate, or three for weak. The withdrawals and dropouts component has an additional ‘not applicable’ option.

The quality assessment checklist also contains the intervention integrity and analysis components, which can be answered from preset options, yes/no, or ‘can’t tell’. The global rating for each paper’s quality was assessed in accordance with the number of components with a weak rating and interpreted as follows: strong (no weak ratings), moderate (one weak rating), or weak (two or more weak ratings). Two authors (SS and YJC) performed a quality assessment. Any discrepancies were resolved by consensus. Funnel plots were used to investigate possible publication bias.

### Data analysis and statistical methods

Primary outcome measures included the proportion of patients screened for metabolic measures, such as weight, glucose, lipids, and BP, and, additionally, ECG, prolactin, liver function tests (LFTs), kidney function, or TSH, in the post-intervention group compared against the pre-intervention group. For secondary outcomes, we characterised each component of institutional intervention programmes implemented to improve metabolic screening rates in hospital-admitted psychiatric patients on antipsychotic treatment. We categorised them per implementing entity, such as provider types, patients, and hospital systems. Intervention components employed in each institution were first qualitatively examined based on the types of implementing entities and methodological strategies. The efficacy of institutional QI initiatives was quantitatively examined by pooling relevant data from each study. Odds ratios (ORs) with 95% confidence intervals (CIs) for metabolic screening events in antipsychotic-treated patients were calculated using the Mantel-Haenszel method, comparing post-interventions to pre-interventions. Either the random- or fixed-effect model was employed in accordance with the level of heterogeneity among the studies included in our meta-analysis. Statistical heterogeneity was examined with χ^2^ statistics, and the *I^2^* statistics was used to quantify inconsistency. *P*<0.1 and *I^2^*>50% suggested statistically significant heterogeneity among the included studies. Two-sided *P*-values were defined as statistically significant if below 0.05. We further conducted sensitivity analyses by excluding low-quality studies due to study designs and risk of confounding, aiming to reduce potential heterogeneity across the included studies. These primary statistical analyses were carried out using Review Manager, version 5.3 (Nordic Cochrane Centre, Cochrane Collaboration, Copenhagen, Denmark). Additionally, a subgroup network meta-analysis was performed to estimate effect sizes of QI intervention components via pooled traditional pair-wise analyses using the ‘netmeta’ package in *R*, version 4.1.0 (R Core Team, Vienna, Austria). Frequentist network meta-analyses were conducted to compare the odds of metabolic monitoring by integrating the direct and indirect effects of each QI intervention strategy, including implementing health care personnel and systemic approaches.

## RESULTS

### Selection of relevant intervention studies

**Figure 1:**
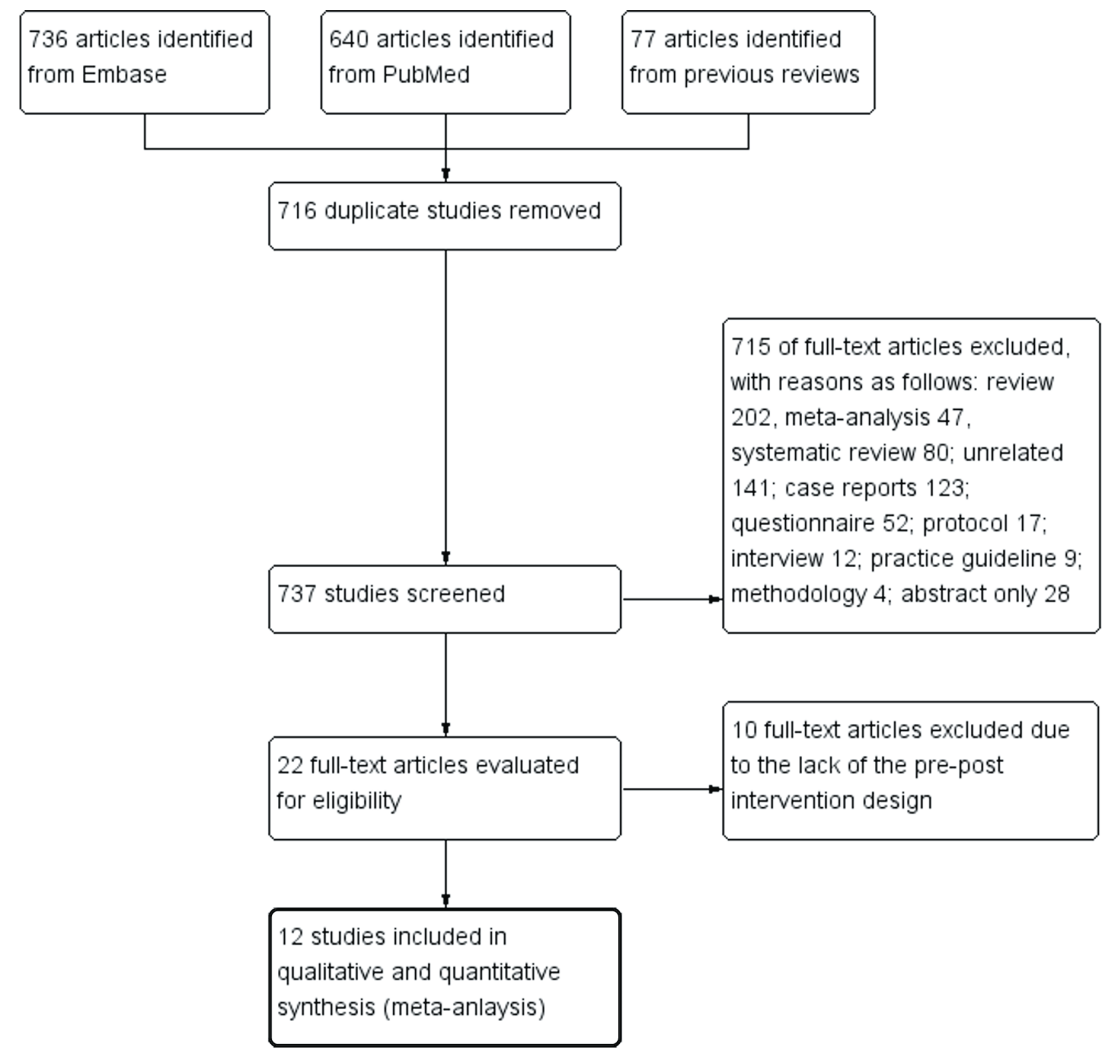
Study selection diagram.

The flow diagram of searching and identifying relevant articles is summarised in **Figure 1**. The initial database search yielded 1453 articles (736, 640, and 77 studies from Embase, PubMed, and previous reviews, respectively), of which 716 duplicates were excluded. The remaining 737 studies underwent further screening of titles and abstracts, resulting in the exclusion of 715 studies. Excluded studies encompassed reviews, meta-analyses, care reports, questionnaires, protocols, among others. After full-text review, 12 studies satisfied the inclusion criteria for qualitative and quantitative analyses, which included a total of 10 128 and 2667 antipsychotic-treated psychiatric patients in the pre- and post-intervention groups, respectively. The 2016 study by Lee et al. [32] is an extended follow-up study of the 2012 study by DelMonte et al. [28]; hence, they were assessed as one study for meta-analysis.

### Study characteristics

We identified 12 intervention studies implemented in inpatient settings aimed at enhancing the screening of metabolic risks in adult mental disease patients maintained on antipsychotic treatment. The characteristics of the 12 studies and overall study findings are summarised in **Table 1** [28,32–42]. All studies employed the pre-post intervention approach in the inpatient settings, with prospective or retrospective audit intervals ranging from two months to four years. The study results in show that metabolic monitoring rates improved by 11.0% and up to 98.0% post-QI interventions, relative to pre-intervention periods. However, only three studies incorporated in their QI programmes follow-up intervention measures in case of abnormal metabolic findings in study patients, such as patient referrals, prescription of medications, change in antipsychotic use, feedback to family doctors and/or patients, and consultation to hospitalists, endocrinologists, or dieticians [32,37,40]. Four studies included only SGA-treated psychiatric patients [28,32,39,41], whereas the remaining eight studies incorporated patients treated with any antipsychotic agents [33–38,40,42]. Five studies were conducted in the United States [28,32,36,37,41], three in the United Kingdom [33,34,38], two in Canada [35,40], and one in Australia [42] and Japan [39]. Four studies reported study patients’ demographic data on age and sex – the mean age was 40.0–55.6 years and 37.9–54.6 years, and the female percentage was 50.0–60.0 and 45.7–57.9% in the pre- and post-intervention groups, respectively [28,32,40,42]. Ethnicity data of study participants were reported in only three studies; less than 20.0% of study participants were non-white in both the pre- and post-intervention groups [28,32,34]. It was assumed that the Japanese study predominantly involved Asian patients, although no information regarding the ethnicity of study participants was available [39].

**Table 1:** Overview of intervention studies to improve metabolic monitoring among antipsychotic-treated patients

Study	Country	Diagnosis/medication	Interventions to improve metabolic monitoring	Study design with intervention duration	Mean age in years*	Sex, % female*	Ethnicity, % non-white*	Sample size (n)*	Interventions to manage metabolic abnormalities	Study results
Runcie et al. 2007 [33]	UK	Severe mental illnesses/any antipsychotics	Protocol was developed and circulated among physicians.	Retrospective audit with three-month follow-up post-intervention				51/61		Monitoring improved inwards with a motivated clinician; overall, monitoring did not improve.
DelMonte et al. 2012 [28]; Lee et al. 2016 [32]	USA	Severe mental illnesses/SGAs only	Physicians were reminded of ordering glucose and lipid levels via electronic pop-up alerts when ordering SGA; a dedicated clinician promoted practice change.	Retrospective audit with up to four-year follow-up post-intervention	43/41	55.0/45.7	20.5/19.4	171/129	Referrals, prescription of medications (Lee et al. 2016 [32])	Pop-up alerts improved ordering rates of glucose and lipid levels from 13–51% at four years; overall rates remain suboptimal.
Ramanuj et al. 2013 [34]	UK	Severe mental illnesses/any antipsychotics	A benchmarked audit of metabolic monitoring, a high-visibility prompt, and an educational programme were provided for physicians.	Prospective audit with up to 13-mo follow-up post-intervention	55.6/54.60	50.0/57.9	2.8/7.9	16/10		Monitoring rates increased from 25–60% post-intervention.
Fischler et al. 2016 [35]	Canada	Schizophrenia, schizoaffective disorder/any antipsychotics	An eight-step framework was developed to enhance the implementation of schizophrenia CPG; CDSS ordered metabolic levels upon antipsychotic prescription; benchmarked feedbacks were provided for clinicians.	Prospective audit with 12-mo follow-up post-intervention				192/184		The percentage of patients monitored for all five metabolic risk factors increased to 56% from 36%.
Kirchner et al. 2016 [36]	USA	Schizophrenia, schizoaffective disorder/any antipsychotics	Implementation of science-informed QIP was developed; clinicians were provided with educational materials, electronic reminders, an audit and feedback on their performance were provided for clinicians; patients in need of metabolic monitoring were identified via EMR review, which was then communicated to a dedicated clinician.	Prospective audit with up to six-month follow-up post-intervention				17/15		Monitoring rates for weight, glucose, and lipids increased after intervention.
Lui et al. 2016 [37]	USA	Severe mental illnesses/any antipsychotics	Implementation of a mandatory admission order set, including four metabolic measures and electronic notification of results.	Retrospective audit with six-month follow-up post-intervention				9100/1499	Change in antipsychotic use, prescription of medications	Monitoring rates for all four metabolic measures increased to 100% from 2% post-intervention.
Green et al. 2018 [38]	UK	Severe mental illnesses/any antipsychotics	A QIP composed of a multi-professional physical health assessment, a patient-held physical health plan, and education and training for staff and patients was implemented; a monitoring tool was developed.	Retrospective audit of a 10-mo baseline period; prospective audit over a 15-mo implementation period				247/318		Improvements were seen in screening for BMI and SBP and in assessment of physical health and CV risk; smoking status was recorded as decreased.
Ishida et al. 2018 [39]	Japan	Psychiatric disorders/SGAs only	A pharmacist-mediated electronic side-effect monitoring tool was developed; based on the protocol prepared, the pharmacist requested physicians to order clinical laboratory tests.	Retrospective audit with up to 12-mo follow-up post-intervention				132/143		Implementation rates of clinical laboratory tests post-intervention increased from 80–91%.
Ross et al. 2018 [40]	Canada	Psychotic disorders, mood disorders, Substance use, posttraumatic stress disorder, adjustment disorder, dementia, delirium/any antipsychotics	A standard electronic admission order set and training to inpatient clinical staff were implemented.	Retrospective audit with three-month follow-up post-intervention	42.0/42.5	53.0/52.0		96/190	Feedback to family doctor and/or patient, consultation to hospitalist, endocrinology, and/or dietician	Significant improvements were observed in monitoring rates for blood glucose (from 31–96%), lipids (from 36–64%), ECG (51–87%), and TSH (from 71–86%).
Leung et al. 2021 [41]	USA	SGA only	A pharmacist CPA was implemented as part of a QIP.	Retrospective audit with six-month follow-up post-intervention						Compliance of SMD monitoring increased from 69–90%.
Viglione et al. 2021 [42]	Australia	Any antipsychotics	An education campaign for mental health workers was implemented.	Retrospective audit with two-month follow-up post-intervention	40.0/37.9	60.0/57.0		106/118		Monitoring rates for lipids increased from 22–79% and blood glucose from 21–74%; absolute compliance rates remain low.

BMI – body mass index, CDSS – clinical decision support system, CPA – collaborative practice agreement, CPG – clinical practice guideline, CV – cardiovascular, ECG – electrocardiogram, EMR – electronic medical record, QIP – quality improvement programme, SBP – systolic blood pressure, SGAs – second-generation antipsychotics, SMD – screening for metabolic disorder, TSH – thyroid stimulating hormone

*Values are presented pre-/post-intervention.

Each study site implemented varying types of QI strategies targeting provider groups, patients, or institutional systems (**Table 2**). In 10 out of the 12 studies, physicians were the key players in implementing the institutional QI programmes with the support of education, individual audit or feedback, and metabolic screening tools or prompts [28,32–36,38–40,42]. Of the individual components of intervention strategies, 11 intervention programmes incorporated systemic approaches in their QI programmes, ranging from protocol development, patient identification and communication to family physicians via the electronic medical record (EMR) system and electronic pop-up alerts to designation of champion psychiatrists, standardised electronic order sets and barrier assessment [28,32–41]. Pharmacists and nurses also contributed to the institutional QI programmes as moderators or patient educators in four [28,32,39,41] and three studies [38,40,42], respectively. Patient engagement strategies, such as incorporating patient education and feedback, were employed in three studies [35,38,40]. The number of intervention components employed in the studies ranged from two to eight, with a median of five. The quality appraisal of individual studies on each risk of bias is summarised in Table S1 in the **Online Supplementary Document**. The study design component was evaluated as weak for all included studies due to their non-randomised design. Seven of the studies had a high risk of confounding bias due to the lack of controlled audit cycles and statistical adjustment for potentially confounding patient factors, such as age, sex, and comorbid conditions [33,35–39,41]. Overall, the risk of bias assessment of the included studies was rated moderate to weak. Funnel plots were symmetrical, indicating no publication bias (Figure S1 in the **Online Supplementary Document**).

**Table 2:** Components of QI interventions to improve metabolic monitoring for antipsychotic-treated patients by implementing entities*

	Physician			Pharmacist	Nurse	Patient	System						
**Study**	**Education**	**Individual audit/feedback**	**Metabolic screening tool/prompts**	**Mediation**	**Education/mediation**	**Education/feedback**	**Protocol/leadership support**	**Patient identification**	**Communication to family physician**	**Electronic pop-up alert**	**Champion psychiatrist**	**Standardised electronic order set**	**Barrier assessment**
Runcie et al. 2007 [33]	V		V				V						
DelMonte et al. 2012 [28]; Lee et al. 2016 [32]	V		V	V						V	V		
Ramanuj et al. 2013 [34]	V	V	V				V			V			V
Fischler et al. 2016 [35]	V	V	V			V	V			V		V	V
Kirchner et al. 2016 [36]	V	V					V	V		V	V		V
Lui et al. 2016 [37]								V		V		V	
Green et al. 2018 [38]	V	V	V		V	V	V				V		V
Ishida et al. 2018 [39]			V	V			V	V		V			
Ross et al. 2018 [40]	V	V	V		V	V			V			V	
Leung et al. 2021 [41]				V			V						
Viglione et al. 2021 [42]	V				V								

QI – quality improvement

*The letter ‘V’ indicates that the given QI programme incorporated the specific intervention strategies.

### The efficacy of institutional interventions on metabolic monitoring

**Figure 2:**
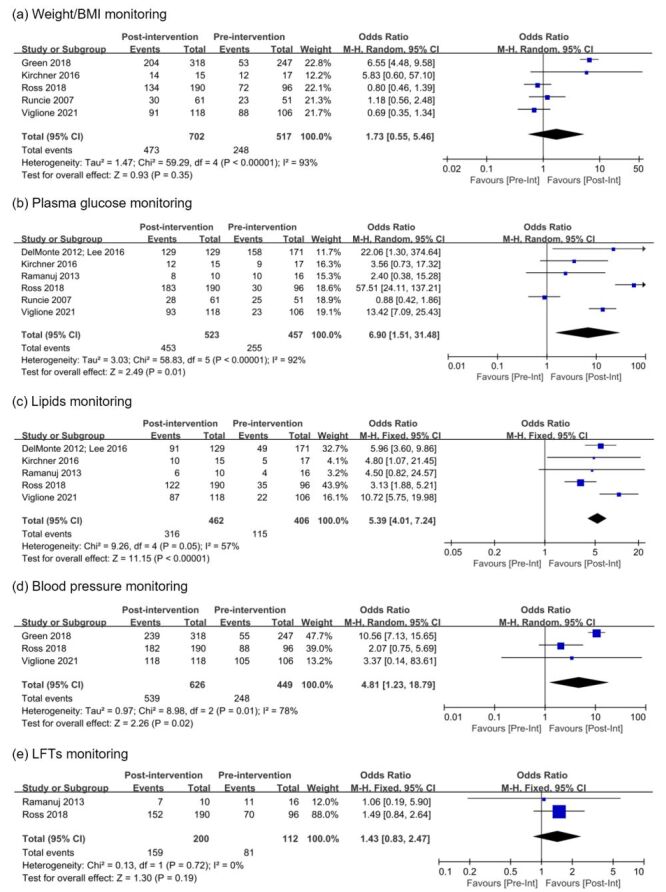
Forest plots for meta-analysis of odds ratios examining the effects of institutional QI interventions on antipsychotic-associated metabolic risk monitoring in hospitalised patients with mental disorders. BMI – body mass index, CI – confidence interval, LFTs – liver function tests, M-H – Mantel-Haenszel, QI – quality improvement

Individual cardiometabolic measures screened for antipsychotic-treated patients in each intervention study are summarised in **Table 3**, which include weight, BMI, waist circumference, BP, ECG, blood glucose, lipids, prolactin, LFTs, kidney function, and TSH. The most commonly monitored cardiometabolic measures were blood glucose (11 studies) [28,32–38,40–42], lipids (nine studies) [28,32,34–37,40–42], weight/BMI/waist circumference (seven studies) [33,35–38,40,42], and BP (five studies) [35,37,38,40,42]. Nine out of 12 studies, or 75.0% of studies, reported changes in monitoring rates for both blood glucose and lipids before and after QI interventions [28,32,34–37,40–42]. Only four out of 12 studies, or 33.3%, reported screening rates for all four metabolic parameters (plasma glucose, lipids, weight/BMI, and BP) [35,37,40,42]. The efficacy of institutional QI programmes in improving screening rates for major cardiometabolic measures was quantitatively assessed, and the results are graphically summarised with forest plots in **Figure 2**. Monitoring rates for plasma glucose, lipids, and BP were substantially increased following QI interventions, with the OR = 6.90 (95% CI = 1.51–31.48), OR = 5.39 (95% CI = 4.01–7.24), and OR = 4.81 (95% CI = 1.23–18.79), respectively. In the sensitivity analysis, the odds of monitoring for glucose and lipids remained statistically significant after excluding studies of low quality and high heterogeneity in terms of study designs and confounding risks, with OR = 16.02 (95% CI = 4.67–54.92) and OR = 5.41 (95% CI = 4.00–7.32), respectively (Figure S2 in the **Online Supplementary Document**). When we quantitatively calculated the proportion of patients screened after pooling monitoring rates from each study, the median metabolic screening rates for plasma glucose, lipids, and BP increased from 51.0–80.0%, from 28.7–66.7%, and 91.7–95.8%, respectively. The ORs for weight/BMI and LFTs monitoring also suggested a positive impact of QI programmes, albeit not associated with statistical significance.

**Table 3:** Metabolic parameters monitored for antipsychotic-treated patients in each intervention study*

Laboratories	Weight	BMI	Waist circumference	Blood pressure	ECG	Blood glucose	Lipids	Prolactin	LFTs	Kidney function	TSH
Runcie et al. 2007 [33]	V					V					
DelMonte et al. 2012 [28]; Lee et al. 2016 [32]						V	V				
Ramanuj et al. 2013 [34]					V	V	V	V	V	V	V
Fischler et al. 2016 [35]	V	V	V	V		V (HbA1c)	V				
Kirchner et al. 2016 [36]	V					V	V				
Lui et al. 2016 [37]	V	V	V	V		V	V				
Green et al. 2018 [38]	V	V		V							
Ishida et al. 2018 [39]						V					
Ross et al. 2018 [40]	V	V	V	V	V	V	V	V	V	V	V
Leung et al. 2021 [41]						V	V				
Viglione et al. 2021 [42]	V	V	V	V		V	V				

BMI – body mass index, ECG – electrocardiogram, HbA1c – glycated haemoglobin, LFTs – liver function tests, TSH – thyroid stimulating hormone

*The letter ‘V’ indicates that the given QI programme incorporated the specific intervention strategies.

**Figure 3:**
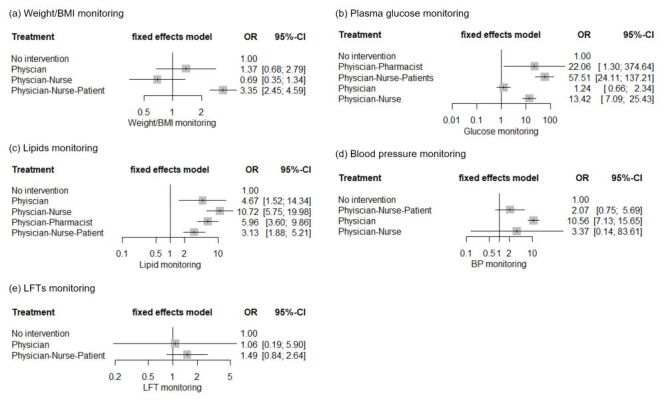
Forest plots for network meta-analysis of odds ratios examining the effects of QI strategies per targeting entity (physician, pharmacist, nurse, and patient) on antipsychotic-associated monitoring risk monitoring in hospitalised patients with mental disorders. BMI – body mass index, CI – confidence interval, LFTs – liver function tests, M-H – Mantel-Haenszel, QI – quality improvement

In our subgroup network meta-analysis, the odds of monitoring for weight/BMI and glucose were highest when physicians, nurses, and patients were all engaged in QI programmes, resulting in OR = 3.35 (95% CI = 2.45–4.59) and OR = 57.51 (95% CI = 24.11–137.21), respectively (**Figure 3**). QI interventions involving multidisciplinary health care professionals demonstrated substantially improved lipids monitoring rates. Physician involvement significantly increased the rate of BP monitoring (OR = 10.56; 95% CI = 7.13–15.65). However, no significant changes in LFT monitoring rates were observed with any QI interventions per participating entity. Another subgroup analysis investigating the impact of specific systemic strategies adopted for QI interventions on cardiometabolic monitoring showed insignificant odds of monitoring rates compared to no intervention (Figure S3 in the **Online Supplementary Document**). Most QI programmes promoting physician engagement also incorporated multiple systemic approaches. These results suggest that the engagement of multidisciplinary professionals and patients may be more crucial than the specific types of systemic approaches adopted for QI intervention in improving cardiometabolic monitoring rates in hospitalised patients on antipsychotic agents.

## DISCUSSION

SMI patients face an increased risk of diabetes, metabolic syndrome, CV complications, and shorter life expectancy relative to non-psychiatric people [1,2,43–45]. In addition to these risks, the metabolic adverse effects of antipsychotics further increase the vulnerability of these patients to cardiometabolic complications; therefore, periodic risk monitoring is crucial to improving prognostic outcomes in these high-risk patients [11–13,46,47]. Of SGAs commonly associated with metabolic abnormalities, clozapine and quetiapine pose severe risks, contributing to both weight gain and diabetes/dyslipidaemia. Quetiapine carries an intermediate risk for weight gain and a significant risk for diabetes or dyslipidaemia, while risperidone and paliperidone present an intermediate risk for weight gain [7,8]. Among first-generation agents, chlorpromazine poses a significant risk for both weight gain and diabetes or worsening lipid profile, thioridazine and haloperidol show an intermediate risk for weight gain, and thioridazine also demonstrates a significant risk for diabetes or lipid abnormalities [7,8]. The recommended monitoring parameters according to the 2004 consensus statement released by the American Diabetes Association and the American Psychiatric Association are as follows. For baseline, it is advised to assess personal and family history, weight and height, waist circumference, BP, fasting plasma glucose, and fasting lipid profile [11]. During follow-up, weight reassessment is recommended at intervals such as four, eight, and 12 weeks, followed by quarterly evaluations [11]. Additionally, glucose, lipids, and blood pressure should be reassessed at three months and annually thereafter. For those with a normal lipid profile, it is advisable to consider repeating testing at five-year intervals [11]. Patients treated with SGAs or high-risk antipsychotics may warrant more frequent monitoring if clinically indicated [11].

For guideline-concordant practice, all patients in receipt of antipsychotic treatment should be monitored regularly for possible metabolic adverse effects. Previous observational studies, however, showed that, in real-world practice, antipsychotic-associated risk screening fell short of guideline recommendations [20–22]. By combining the effects of guideline dissemination reported in these observation studies, a 2012 meta-analysis examined the efficacy of passive uptake of guideline recommendations [23]. The meta-analysis demonstrated that metabolic risk screening remained suboptimal and performed in less than 50.0% of patients – BP and triglycerides were the only parameters whose monitoring rates were greater than 50.0% [23]. These findings spurred a series of intervention studies where QI programmes were developed and implemented to enhance metabolic risk monitoring in antipsychotic-receiving patients [24–26]. Practice audits were performed before and after the implementation of interventions, and the post-intervention audit revealed substantially improved screening but persistent suboptimal rates of metabolic recording [24–26]. A 2019 systematic review examined the quality of interventions designed for either outpatient or hospital-admitted patient monitoring and evaluated in a descriptive manner the efficacy of those interventions in improving risk monitoring rates among antipsychotic-treated patients [27]. Overall, the median metabolic screening rates for all four measures (glucose, lipids, obesity, and BP) increased from 11.0% in the comparison group to 57.0% in the intervention group [27]. However, the authors noted that substantial heterogeneity across the included intervention studies precluded them from quantitatively combining the efficacy results from each study to perform meta-analysis [27]. Nevertheless, they suggested that the interventions were effective in narrowing the guideline-practice gap, although up to one-third of patients left unscreened [27]. Some intervention studies assessed barriers to guideline adherence, including practical issues, such as lack of adequate resources or training to monitor clinics [25,36,48,49]. Difficulties in obtaining fasting blood samples and performing on-site testing could also contribute to suboptimal guideline adherence in outpatient or community settings [25,49].

In our meta-analysis, we focused on institutional QI interventions implemented in hospitals and designed for hospital-admitted psychiatric patients in receipt of antipsychotic therapy. Inpatient care settings provide an advantageous opportunity in that interventions are less likely to be challenged by the aforementioned barriers in terms of limited resources and training [40]. In addition, due to the observed connection between a history of hospital admission and increased mortality in psychiatric patients, achieving a positive impact is more attainable through interventions focused on high-risk inpatients within hospital settings [50]. The studies included in our analysis are characterised by certain shared properties, such as the implementation of intervention programmes to enhance metabolic risk monitoring in antipsychotic-treated patients, inpatient settings, and the pre-post comparison design. These shared properties helped to decrease heterogeneity concerning study design, setting, outcomes, and intervention strategies across the included studies. To the best of our knowledge, this was the first study to perform meta-analysis to synthesise quantitative evidence on the efficacy of QI interventions on improving antipsychotic-associated risk monitoring in inpatient settings. Inpatient care offers an opportunity not only to perform comprehensive metabolic risk screening but also to intervene with follow-up measures to treat or manage identified metabolic risks. The current meta-analysis suggests that institutional QI programmes were substantially effective in increasing metabolic monitoring rates in antipsychotic-treated mental disease patients. Although the focus of the current study has been the four metabolic parameters (glucose, lipids, weight/BMI, and BP), expanding the scope to include additional metabolic parameters, such as prolactin, TSH, and ECG, could offer a more comprehensive understanding of the cardiometabolic risks associated with antipsychotic medications. Of the individual studies included in this meta-analysis, only 33.3% (4/12) studies incorporated follow-up clinical interventions in case of abnormal metabolic findings in patients, such as referral arrangements, switch or prescription of medications, consultation with other clinicians, and communication to primary care providers [32,37,40]. The crucial aspect, where the greatest benefits for patients would arise, involves addressing the abnormal metabolic findings in these individuals. We can adopt strategies commonly utilised in previous intervention studies. Moreover, beyond the dedication of the leading multidisciplinary team, leadership support and systemic assistance that facilitate information sharing, and reduce the labour and time burden on health care team members, should bolster the QI initiative.

The current study identified key components of institutional QI programmes to improve metabolic risk screening and, more importantly, to facilitate medical follow-up in case of abnormal findings being identified in antipsychotic-treated inpatients. Most QI programmes employed a multifaceted approach by incorporating diverse intervention strategies. Of those, the strategies implemented at the level of physicians were central in many QI programmes, such as the provision of physician education and reminders, individual audit/feedback, and metabolic screening tools/prompts [28,32–36,38–40,42]. Another essential component in QI programmes was organisational involvement or hospital system-based approach, such as protocol development, leadership support and commitment, utilisation of electronic pop-up alerts and standardised order set, designation of a champion clinician for metabolic monitoring, and barrier identification [28,32–41]. In addition, engaging patients in their health care by providing education and feedback [35,38,40] along with improving communication with primary care providers [40] can further promote persistent adherence in antipsychotic-associated risk screening and have a positive impact on long-term outcomes of these QI programmes. Patient-centred care entails actively involving patients in their disease management and health care decisions, fostering a collaborative relationship between health care professionals and patients. patient engagement becomes crucial, as individuals with mental disorders may face distinct challenges in understanding and managing their conditions. Pharmacists, as integral members of the health care team, can contribute significantly to patient-centred approaches as they are well-positioned to offer patient counselling and feedback. This involves not only conveying information on the therapeutic benefits of antipsychotic medications but also addressing potential side effects. Specifically, the discussion encompasses how these side effects might elevate the risk of cardiometabolic complications if not adequately addressed. This approach aims to empower patients, address potential challenges unique to mental disorder inpatients, and ultimately improve the effectiveness of antipsychotic-associated metabolic risk management.

In QI efforts, sustainability is a vital consideration that merits careful planning, and this is where delegating tasks to non-physician health care professionals, such as pharmacists and nurses, becomes essential for achieving sustainable success of institutional intervention programmes. Our network meta-analysis showed that the most substantial impact on metabolic risk monitoring was achieved when multidisciplinary health care professionals and patients were collectively engaged in the QI efforts. In the study by Ishida et al., a protocol based on the guidelines was established under which clinical pharmacists could request clinical laboratory orders at appropriate times [39]. They also developed a pharmacist-mediated management tool for side effect monitoring using database software, which enabled efficient information sharing and collaboration between multidisciplinary health professionals, centralised monitoring activities, and reduced health care professionals’ work hours and labour [39]. Incorporating metabolic monitoring templates for pharmacists and nursing notes in the EMR system can enhance the efficiency of the monitoring process. Clinical pharmacists can also provide pharmacotherapy education for clinicians and patients and therapeutic recommendations for the management of identified metabolic risks [51]. Nurses establish close relationships with patients, offering care and, as a result, can deliver patient education, conduct assessments, and make referrals to pertinent health care providers [52]. Implementing nurse-mediated institutional QI programmes played a role in standardising care provision and enhancing professional practice [53,54].

This study has some limitations. First, as this study is focused on hospitalised patients, which limits the generalisability of the results, our findings may not be applicable to other settings, such as outpatient care. Second, in order to decrease heterogeneity across the included studies, we restricted eligible studies to those with the following specific attributes, thereby limiting the number of studies that met the criteria – pre-post intervention design, institutional QI programmes, and inpatient settings. The overall quality of the intervention studies included in this meta-analysis was rated as weak or moderate, with none receiving a strong rating. However, the nature of the pre-post intervention design inherently introduces limitations, as these studies are primarily non-randomised. Furthermore, the reliance on descriptive statistics for reporting study results poses challenges in performing robust statistical adjustments for confounding factors. Given the constraints imposed by the study designs and the nature of reported results, a certain level of methodological limitation is inevitable. Furthermore, each study incorporated multiple improvement strategies with varying audit intervals and had methodological limitations, such as non-randomised controlled design and no adjustment for confounding factors, which could negatively affect our ability to infer causality and make definitive conclusions about the actual effectiveness of the QI programmes. However, given the multifaceted nature of QI programmes and the diverse resources available in hospitals, assessing the impact of institutional QI initiatives through randomised, controlled designs presents a challenge. Nevertheless, the overall quality of the included studies, with a high risk of confounding bias, and the wide range of intervention strategies could all negatively impact the robustness and generalisability of our findings. Individual studies exhibited significant diversity in their focus on enhancing specific metabolic measures. Some studies concentrated on a single parameter, such as glucose, whereas others tackled a broader spectrum, encompassing glucose, lipids, weight, and BP. SMI patients are vulnerable to abnormalities across all these metabolic parameters. Therefore, future surveillance initiatives related to antipsychotics should be designed in alignment with established guidelines, aiming to establish more comprehensive monitoring of cardiometabolic risks. Despite the aforementioned limitations, the current study holds clinical significance in that it was the first meta-analysis quantitatively evaluating the impact of institutional QI programmes on monitoring rates in hospitalised patients with mental disorders treated with antipsychotics.

## CONCLUSIONS

Our study findings demonstrated that institutional QI interventions were effective in filling the guideline-practice gap in monitoring antipsychotic-induced metabolic side effects in inpatient settings. However, up to two-thirds of the intervention studies lacked or did not report on follow-up measures to treat or manage identified risks in hospitalised psychiatric patients. Future institutional QI programmes should integrate metabolic risk screening with follow-up risk management measures when risks are identified. In addition to intervention strategies involving physicians and hospital systems, it is crucial to consider delegating tasks to pharmacists and nurses, as well as engaging patients in their health care, to ensure the sustainability of intervention programmes.

## Additional material


Online Supplementary Document
