# Supplementary material for: Impact of institutional quality improvement initiatives on metabolic monitoring in mental disorder in patients treated with antipsychotics: A meta-analysis of intervention studies
Source: J Glob Health. 2024 May 24;14:04074. doi: 10.7189/jogh.14.04074 (PMC11116930; doi:10.7189/jogh.14.04074)
Supplement: Online Supplementary Document [file jogh-14-04074-s001.pdf]

Supplementary material

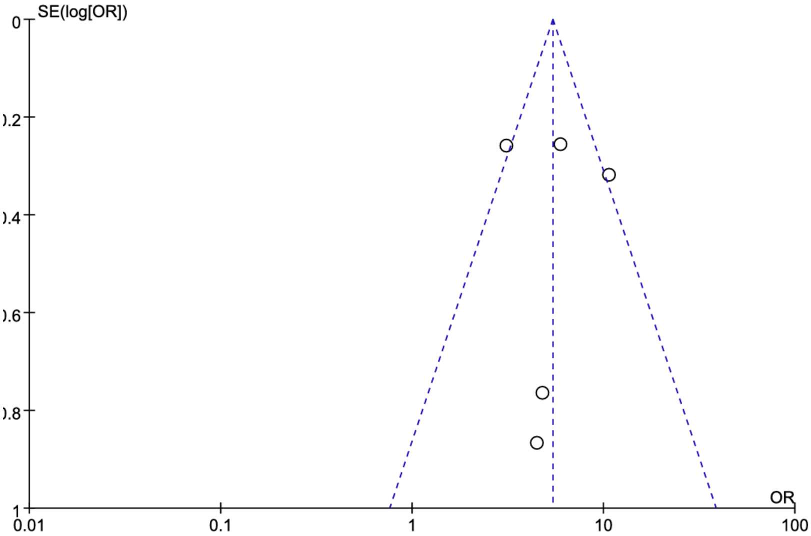

Figure S1. Funnel plot of lipids monitoring.

(a) Plasma glucose monitoring

Study or Subgroup	Post-intervention Events	Post-intervention Total	Pre-intervention Events	Pre-intervention Total	Weight	Odds Ratio M-H, Random, 95% CI
DelMonte 2012; Lee 2016	129	129	158	171	12.7%	22.06 [1.30, 374.64]
Kirchner 2016	12	15	9	17	0.0%	3.56 [0.73, 17.32]
Ramanuj 2013	8	10	10	16	20.5%	2.40 [0.38, 15.28]
Ross 2018	183	190	30	96	32.1%	57.51 [24.11, 137.21]
Runcie 2007	28	61	25	51	0.0%	0.88 [0.42, 1.86]
Viglione 2021	93	118	23	106	34.7%	13.42 [7.09, 25.43]
**Total (95% CI)**		**447**		**389**	**100.0%**	**16.02 [4.67, 54.92]**
Total events	413		221			

Heterogeneity: Tau² = 1.03; Chi² = 12.18, df = 3 (P = 0.007); I² = 75%
Test for overall effect: Z = 4.41 (P < 0.0001)

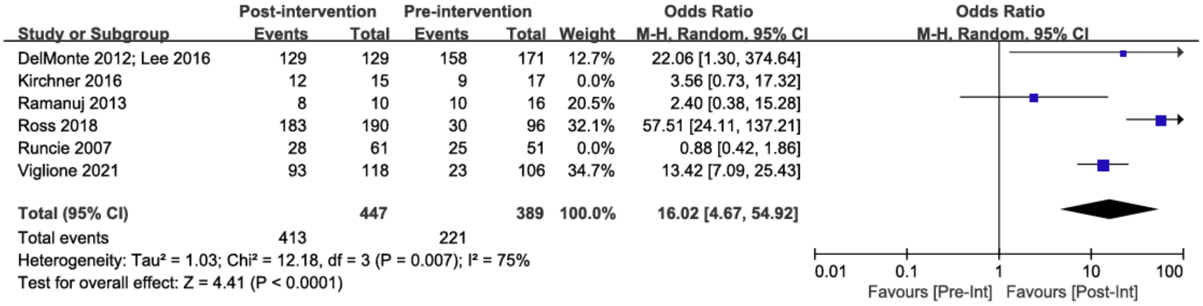

(b) Lipids monitoring

Study or Subgroup	Post-intervention Events	Post-intervention Total	Pre-intervention Events	Pre-intervention Total	Weight	Odds Ratio M-H, Fixed, 95% CI
DelMonte 2012; Lee 2016	91	129	49	171	34.1%	5.96 [3.60, 9.86]
Kirchner 2016	10	15	5	17	0.0%	4.80 [1.07, 21.45]
Ramanuj 2013	6	10	4	16	3.4%	4.50 [0.82, 24.57]
Ross 2018	122	190	35	96	45.8%	3.13 [1.88, 5.21]
Viglione 2021	87	118	22	106	16.7%	10.72 [5.75, 19.98]
**Total (95% CI)**		**447**		**389**	**100.0%**	**5.41 [4.00, 7.32]**
Total events	306		110			

Heterogeneity: Chi² = 9.24, df = 3 (P = 0.03); I² = 68%
Test for overall effect: Z = 10.96 (P < 0.00001)

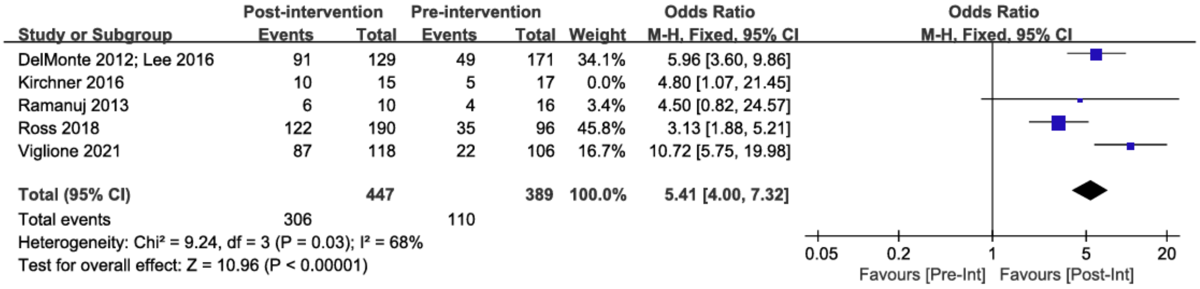

Figure S2. Forest plot of sensitivity analysis of odds ratios examining the effects of institutional QI interventions on antipsychotic-associated metabolic risk monitoring after excluding low quality studies.

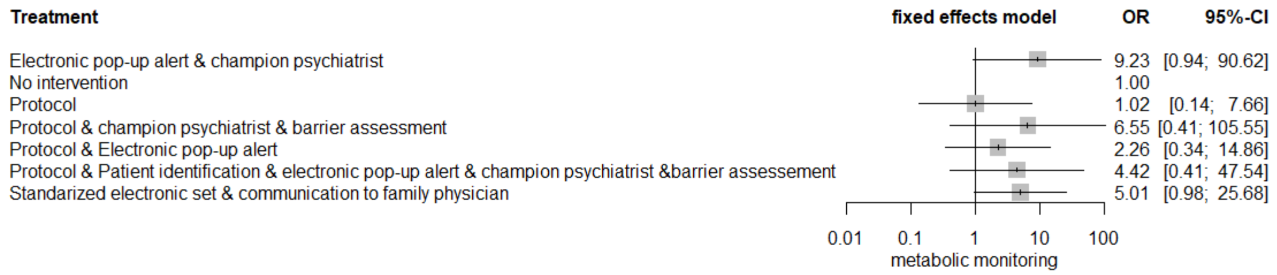

Figure S3. Forest plot for network meta-analysis on the impact of QI system types on cardiometabolic monitoring rates

Table S1. Quality assessment of the intervention studies

Study	Selection bias	Study design	Confounders	Blinding	Data collection method	Withdrawal and drop outs	Global rating
Runcie et al., 2007	1	3	3	2	2	Not applicable	Weak
DelMonte et al., 2012; Lee et al., 2016	1	3	1	2	1	Not applicable	Moderate
Ramanuj et al., 2013	2	3	1	2	1	Not applicable	Moderate
Fischler et al., 2016	1	3	3	3	1	Not applicable	Weak
Kirchner et al., 2016	1	3	3	2	1	Not applicable	Weak
Lui et al., 2016	1	3	3	2	1	Not applicable	Weak
Green et al., 2018	1	3	3	2	1	Not applicable	Weak
Ishida et al., 2018	1	3	3	2	1	Not applicable	Weak
Ross et al., 2018	1	3	1	2	1	Not applicable	Moderate
Leung et al., 2021	1	3	3	2	1	Not applicable	Weak
Viglione et al., 2021	1	3	1	2	1	Not applicable	Moderate

Each of the 6 components (selection, design, confounders, blinding, data collection withdrawal and drop outs) is rated per a standardized guide as 1 (strong), 2 (moderate), or 3 (weak).

Each study is rated with a global rating depending on the number of components with a weak rating. Studies with no weak components are rated strong, studies with 1 weak component are rated moderate and studies with 2 or more weak components are rated as weak.
